# Electrografting a Hybrid Bilayer Membrane via Diazonium Chemistry for Electrochemical Impedance Spectroscopy of Amyloid-β Aggregation

**DOI:** 10.3390/mi13040574

**Published:** 2022-04-05

**Authors:** Hamid Fini, Qusai Hassan, Meissam Noroozifar, Kagan Kerman

**Affiliations:** Department of Physical and Environmental Sciences, University of Toronto Scarborough, 1265 Military Trail, Toronto, ON M1C 1A4, Canada; h.fini@utoronto.ca (H.F.); qusai.hassan@mail.utoronto.ca (Q.H.); m.noroozifar@utoronto.ca (M.N.)

**Keywords:** Hybrid bilayer membrane, Diazonium chemistry, electrodeposition, amyloid-β

## Abstract

Herein, a novel hybrid bilayer membrane is introduced as a platform to study the aggregation of amyloid-β_1–42_ (Aβ_1–42_) peptide on surfaces. The first layer was covalently attached to a glassy carbon electrode (GCE) via diazonium electrodeposition, which provided a highly stable template for the hybrid bilayer formation. To prepare the long-chain hybrid bilayer membrane (lcHBLM)-modified electrodes, GCE surfaces were modified with 4-dodecylbenzenediazonium (DDAN) followed by the modification with dihexadecyl phosphate (DHP) as the second layer. For the preparation of short-chain hybrid bilayer membrane (scHBLM)-modified electrodes, GCE surfaces were modified with 4-ethyldiazonium (EDAN) as the first layer and bis(2-ethylhexyl) phosphate (BEHP) was utilized as the second layer. X-ray photoelectron spectroscopy (XPS) and time-of-flight secondary ion mass spectrometry (ToF-SIMS) were used to characterize the bilayer formation. Both positively charged [Ru(NH_3_)_6_]^3+^ and negatively charged ([Fe(CN)_6_]^3-/4-^) redox probes were used for electrochemical characterization of the modified surfaces using cyclic voltammetry (CV) and electrochemical impedance spectroscopy (EIS). EIS results showed a decrease in charge transfer resistance (R_ct_) upon incubation of Aβ_1–42_ on the hybrid bilayer-modified surfaces. This framework provides a promising electrochemical platform for designing hybrid bilayers with various physicochemical properties to study the interaction of membrane-bound receptors and biomolecules on surfaces.

## 1. Introduction

The direct, specifically in situ, investigation of biological membranes is a challenging topic of intense research interest. To study membrane proteins, scientists need to mimic biomembranes using various approaches to stabilize the bilayers on a solid substrate surface [1]. A wide variety of biomimetic membranes have been developed in the past [1,2,3,4,5,6,7,8,9,10]. Synthetic bilayer membranes made of lipids have been broadly employed as models to study biomembranes since they were first introduced in the form of black lipid membranes by Mueller et al. in 1962 [11]. The formation of biomembrane-like bilayer structures from a simple organic compound was introduced by Kunitake and Okahata in 1977 [12]. The most commonly reported types of biomimetic membranes are free solid-supported membranes, [7,8,13,14,15], polymer-cushioned membranes [16,17], tethered bilayer membranes [18,19], and hybrid bilayer membranes [9,10,20,21,22,23]. Hybrid bilayer lipid membranes (HBLMs) are often composed of a thiolated-alkyl chain covalently attached to a gold surface, resulting in the formation of a self-assembled monolayer (SAM), while the second layer is typically composed of a phospholipid [3,9,24]. The covalent nature of the first layer of the HBLMs provides stability against changes in the pH, ionic strength, and composition of buffer solutions [22,25,26]. HBLMs display significantly more mechanical stability compared to other types of bilayers [7,23]. Because of their rugged formation on the surface, HBLMs can be studied using a wide variety of analytical techniques, such as surface plasmon resonance [25,26], infrared spectroscopy [27], neutron reflectometry [24,27,28], vibrational spectroscopy [29], surface enhanced Raman scattering [30,31], spectroelectrochemistry [32], cyclic voltammetry (CV) [20,21,33,34,35,36,37], and electrochemical impedance spectroscopy (EIS) [33,35,38,39]. EIS is particularly advantageous as it provides the ability to study the surface-controlled processes with high sensitivity while determining the charge-transfer resistance (R_ct_) and double-layer capacitance (C_dl_) along with diffusing species as represented by the Warburg element (Z_W_). Furthermore, when using EIS, there is no need for conjugation of analytes (e.g., amyloid-β_1–42_ (Aβ_1–42_) peptides) with a redox-active label [40].

HBLMs have been prepared on different substrates. Whereas HBLMs have traditionally been developed on gold surfaces with thiol alkyl modifications [7,10,21,36,41], carbon ones are yet to be extensively applied to construct HBLMs [42,43,44,45] despite the fact that they provide a relatively inert and stable surface [46]. Diazonium salts are excellent candidates for the covalent modification of carbon surfaces [47,48,49,50,51,52,53,54,55]. One of the earliest examples of using diazonium salts to electrochemically modify glassy carbon electrodes (GCE) was reported by Bélanger et al. [48], who electrodeposited the diazonium analogues of 4-nitrophenyl and 4-carboxyphenyl. Szunerits et al. [56] functionalized GCEs with diazonium salts in ionic liquids. Researchers have also shown that following the electrodeposition of aryl diazonium moieties, further post-functionalization modifications can be attained [55,57]. Several researchers also developed biosensors that incorporated diazonium modifications [45,58,59,60].

Amyloid-β (Aβ) is a hallmark protein implicated in Alzheimer’s disease (AD). The ability of Aβ peptides to disrupt membrane integrity have been studied by numerous research groups in the past decade [61,62,63,64]. Lindberg et al. [65] reported that lipid membranes of dioleoylphosphatidylcholine catalysed the fibril formation of Aβ_1–42_ through lipid–fibril interactions that reinforced secondary pathways. Single molecule microscopy has been applied to track individual Aβ peptide diffusion on lipid bilayers, with Chang et al. [66] reporting that trimers and larger oligomers were immobilized on the lipid bilayer. Kandel et al. [67] studied the cholesterol-dependent membrane pore formation of Aβ_25–35_ peptides. Additional evidence was provided by Capone et al. [68] suggesting that Aβ peptides induced an ion channel-like ion flux in cellular membranes that was independent from the postulated ability of Aβ to modulate intrinsic ion channels or transporter proteins. Lal et al. [69] reviewed the high-resolution 3D structure of Aβ channels and their relevance to the amyloid channel paradigm. Recently, it has been established that Aβ oligomers had a profound detergent-like effect on lipid membrane bilayers as imaged by AFM and electron microscopy. Since the aggregation of Aβ peptides has been a topic of significant interest, there have been numerous studies to follow this process using electrochemical techniques [70,71,72,73,74,75,76,77,78,79,80]. 

In this study, diazonium salts have been employed to prepare HBLM as a platform for electrochemical studies. The first and the second layers of HBLM were characterized by time-of-flight secondary ion mass spectrometry (ToF-SIMS), contact angel measurements, CV, and EIS. This platform was then used to study the aggregation of Aβ_1–42_, using EIS in connection with a positively charged redox probe, ruthenium hexamine, [Ru(NH_3_)_6_]^3+^, and a negatively-charged one, potassium ferri/ferrocyanide, [Fe(CN)_6_]^3-/4-^. Our results demonstrated that long-chain HBLM (lcHBLM) provided a promising platform for studying Aβ_1–42_ aggregation towards a wide range of drug screening studies targeting Aβ_1–42_ oligomers.

## 2. Materials and Methods

### 2.1. Chemicals

HBF_4_ (48% in H_2_O), 4-dodecylaniline (97%), 4-ethylaniline (98%), NaNO_2_ (97.0%), acetic acid (≥99.7%), propionic acid (≥99.5%), acetonitrile (≥99.9%), tetrabutylammonium tetrafluoroborate (>99%), dihexadecyl phosphate (DHP), bis(2-ethylhexyl) phosphate (BEHP), potassium hexacyanoferrate(III) K_3_[Fe(CN)_6_], potassium hexacyanoferrate (II) trihydrate, K_2_[Fe(CN)_6_], (≥99.5%), 1,1,1,3,3,3-hexafluoro-2-propanol (HFIP, ≥99.9%), ruthenium hexamine [Ru(NH_3_)_6_]^3+^, and phosphate buffered saline (PBS) tablets were obtained from Sigma-Aldrich (Oakville, ON, Canada). Hydrochloric acid (Reagent grade) was obtained from Caledon Laboratories Ltd. (Georgetown, ON, Canada). All solutions were prepared with deionized water obtained with a Millipore system (Woodbine, ON, Canada) after filtration with 0.4 µm filters. Nitrogen gas (99.999% purity) for electrochemical experiments was purchased from Air Liquide (Mississauga, ON, Canada).

### 2.2. Instruments

All electrochemical tests were performed using Autolab Potentiostat/Galvanostat (PGSTAT 302N, Metrohm AG, Herisau, Switzerland) in connection with NOVA™ software (NOVA 2.1.2, Metrohm AG, Herisau, Switzerland). Transmission electron microscopy (TEM) images were obtained using a Hitachi 7500 Transmission Electron Microscope equipped with an Olympus SIS MegaView II 1.35 MB digital camera (Hitachi Ltd., Chiyoda, Tokyo, Japan) and processed by iTEM™ software (iTEM 5.2, Hitachi Ltd., Chiyoda, Tokyo, Japan). Contact angle measurements were performed by using a digital camera (PowerShot S5 IS) (Canon, Tokyo, Japan) and ImageJ software. ToF-SIMS measurements were performed using ToF-SIMS IV (Ion-ToF USA Inc., New York, NY, USA). Ellipsometry measurements were conducted using a M2000V automated variable angle spectroscopic ellipsometer (J.A. Woollam Co. Inc., Lincoln, NE, USA) at the University of Western Ontario. Thermo-Scientific K-Alpha (Mississauga, ON, Canada) was used for XPS experiments. An Agilent 6530 Q-ToF (Santa Clara, CA, USA), Alpha-P FTIR (Bruker, Billerica, MA, USA), and Bruker 500 MHz Ultrashield NMR (Bruker, Billerica, MA, USA) were utilized for chemical characterizations of the synthesized 4-dodecylbenzenediazonium tetrafluoroborate molecule. Bruker Topsin 2.1 and MestreNova 14.2 (Bruker, Billerica, MA, USA) were used to process the NMR data.

### 2.3. Electrode Pretreatment

Glassy carbon electrodes (GCEs) (3.0 mm diameter) were purchased from CHInstruments Inc. (Austin, TX, USA). To prepare the modified electrodes, GCEs were first polished with alumina powder (1, 0.3, and 0.05 µm in sequence), followed by sonication in deionized water and subsequently ethanol (95%) for 30 min each. The electrode surface was then acid-activated by running a cyclic voltammogram in 1 M H_2_SO_4_ for 15 scans between −1.2 and +1.2 V (*vs.* Ag/AgCl) at a scan rate of 0.1 V/s. DHP and BEHP solutions (10 mM) were prepared in deionized water.

### 2.4. Synthesis of 4-dodecylbenzenediazonium tetrafluoroborate (DDAN)

4-Dodecylbenzenediazonium was prepared from 4-dodecylaniline using procedures as reported in the literature for similar molecules [81]. Briefly, 4-dodecylanaline (0.5 g, 2 mmol) was mixed into a solution that contained equal volumes of glacial acetic acid and concentrated propionic acid (7 mL in total), to which 2.5 mL of HBF_4_ was added. The solution was cooled to 6 °C, to which 0.2 g of NaNO_2_ was slowly added. The mixture was stirred for 1 h at 6 °C and was subsequently vacuum filtered to isolate the product, which was washed with ethanol and dried. The yield was measured to be approximately 85.1%. MS (Figure S1), FT-IR (Figure S2), and NMR (Figure S3) spectra of the product are shown in Figures S1–S3, respectively. The isolated product (yellow-orange) was stored in a sealed container over CaCl_2_ at 4 °C until further use.

### 2.5. Synthesis of 4-ethylbenzenediazonium (EDAN)

4-Ethyldiazonium was prepared in situ using a solution of 4-ethylaniline (2 mM) with an equimolar sodium nitrite in 1.25 M HCl solution according to previous reports [54]. This solution was purged with nitrogen gas for at least 10 min.

### 2.6. Electrode Modification

**Figure 1:**
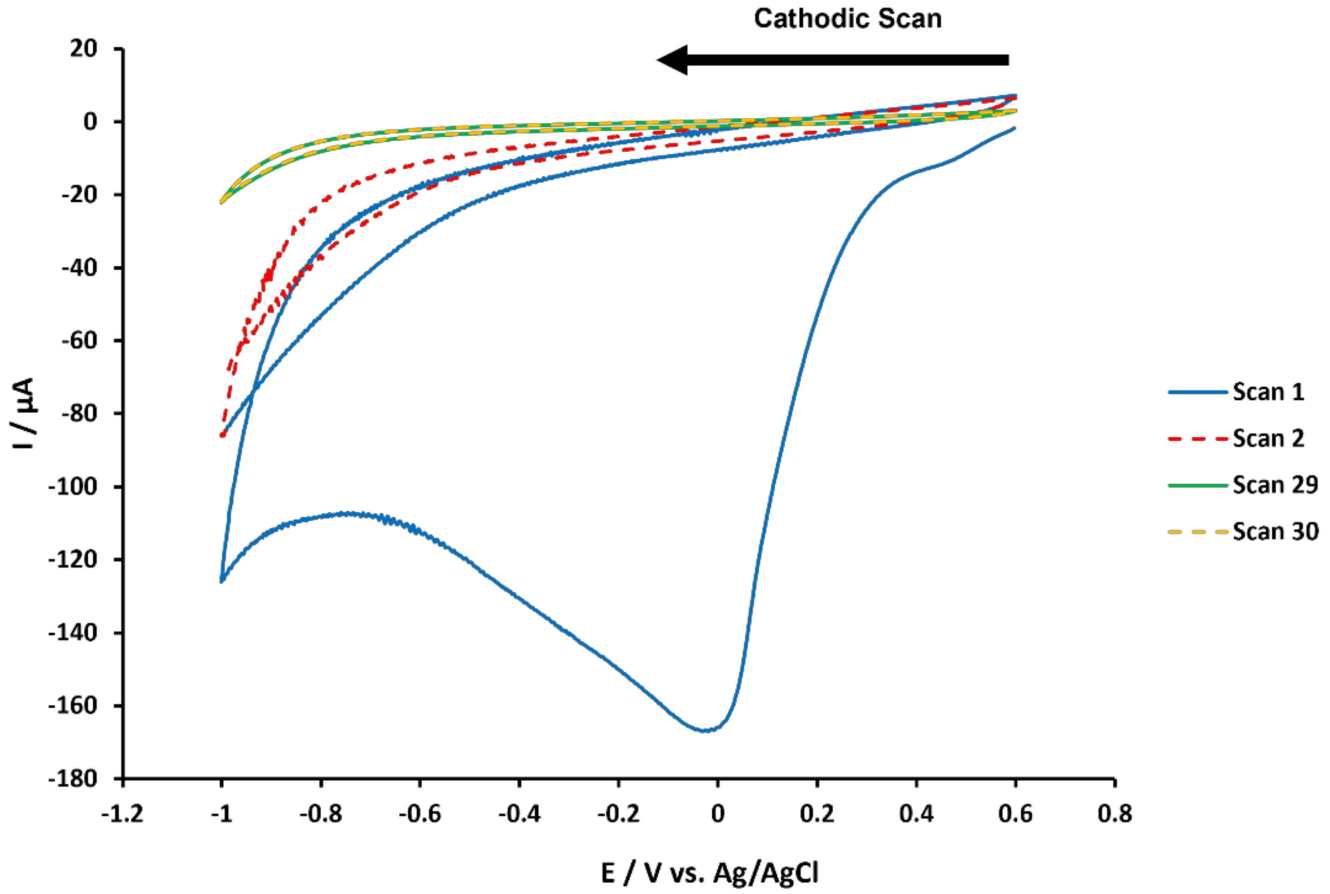
CV of 2 mM DDAN in acetonitrile along with 10 mM of tetrabutylammonium tetrafluoroborate at a scan rate of 0.05 V s^−1^ between +0.6 V and ^_^0.9 V vs. Ag/AgCl: scans 1, 2, 29 and 30 (the last scan) are shown.

For the modification of GCE surfaces with lcHBLMs, GCEs were modified by the 4-dodecylbenzene moiety by first dissolving 4-dodecylbenzene (2 mM) and tetrabutylammonium tetrafluoroborate (10 mM) in acetonitrile. CV was run between −0.9 V and +0.6 V for 30 scans at 50 mV/s (Figure 1), resulting in the formation of the first layer. The electrodes were taken out of the solution, rinsed with deionized water, and sonicated in deionized water for 1 min. For the adsorption of the second layer, a solution of vesicles was prepared by mixing 10 mM DHP in deionized water along with equimolar NaOH to assist dissolution. The solution was then sonicated for 2 h to form a homogenous mixture. Subsequently, GCEs that were formerly modified with the first layer were incubated with 100 µL of the solution overnight. For the modification of GCE surfaces with short-chain hybrid lipid membranes (scHBLMs), immediately following the synthesis of EDAN in situ, CV was applied between 0.6 V and −1.2 V vs. Ag/AgCl for 10 cycles at 50 mV s^−1^ scan rate (Figure S4) [54]. The electrodes were taken out the solution, rinsed with deionized water, and sonicated in deionized water for 1 min. Electrodes modified with 4-ethylbenzene were then incubated with a 10 mM solution of BEHP in deionized water mixed with equimolar NaOH to assist dissolution.

### 2.7. Electrochemical Measurements

The modified GCEs (both ethyl and dodecyl bilayers) were analyzed electrochemically using cyclic voltammetry (CV) and electrochemical impedance spectroscopy (EIS) in either a 2.5 mM solution of [Fe(CN)_6_]^3-/4-^ with 50 Mm NaBr or a 2 mM solution of [Ru(NH_3_)_6_]^3+^ with 50 mM NaBr (NaBr was added to the aforementioned solutions to act as a counterion). CV measurements were performed at a scan rate of 50 mV s^−1^ unless otherwise stated. EIS data were collected as Nyquist plots with an applied bias of −0.2 V and +0.2 V for measurements performed using [Ru(NH_3_)_6_]^3+^ and [Fe(CN)_6_]^3-/4-^, respectively at a frequency ranging from 100 MHz to 100 kHz.

### 2.8. Aβ_1–42_ Aggregation Studies

Aβ_1–42_ was obtained from AnaSpec Inc. (Fremont, CA). Aβ_1–42_ was first treated using HFIP as described before [82]. Briefly, 0.5 mL of HFIP was added to 1 g of Aβ_1–42_, which was then sonicated for 15 min. The solution was then stored at 4°C overnight. The solution was then aliquoted, after which the HFIP was dried under N_2_. Aβ_1–42_ films were then stored at −20 °C until required for the experiment. Immediately before measurement, 1 mL of 0.01 M phosphate buffer (pH 7.4) was added to dissolve the peptide. lcHBLM-modified GCEs were then incubated with 100 µL of the peptide solution for 10 min, 24 h, and 48 h. Aβ_1–42_-modified lcHBLM surfaces were gently washed with deionized water immediately before EIS measurements.

## 3. Results and Discussion

### 3.1. Covalent Modification of GCE Surfaces

**Scheme 1:**
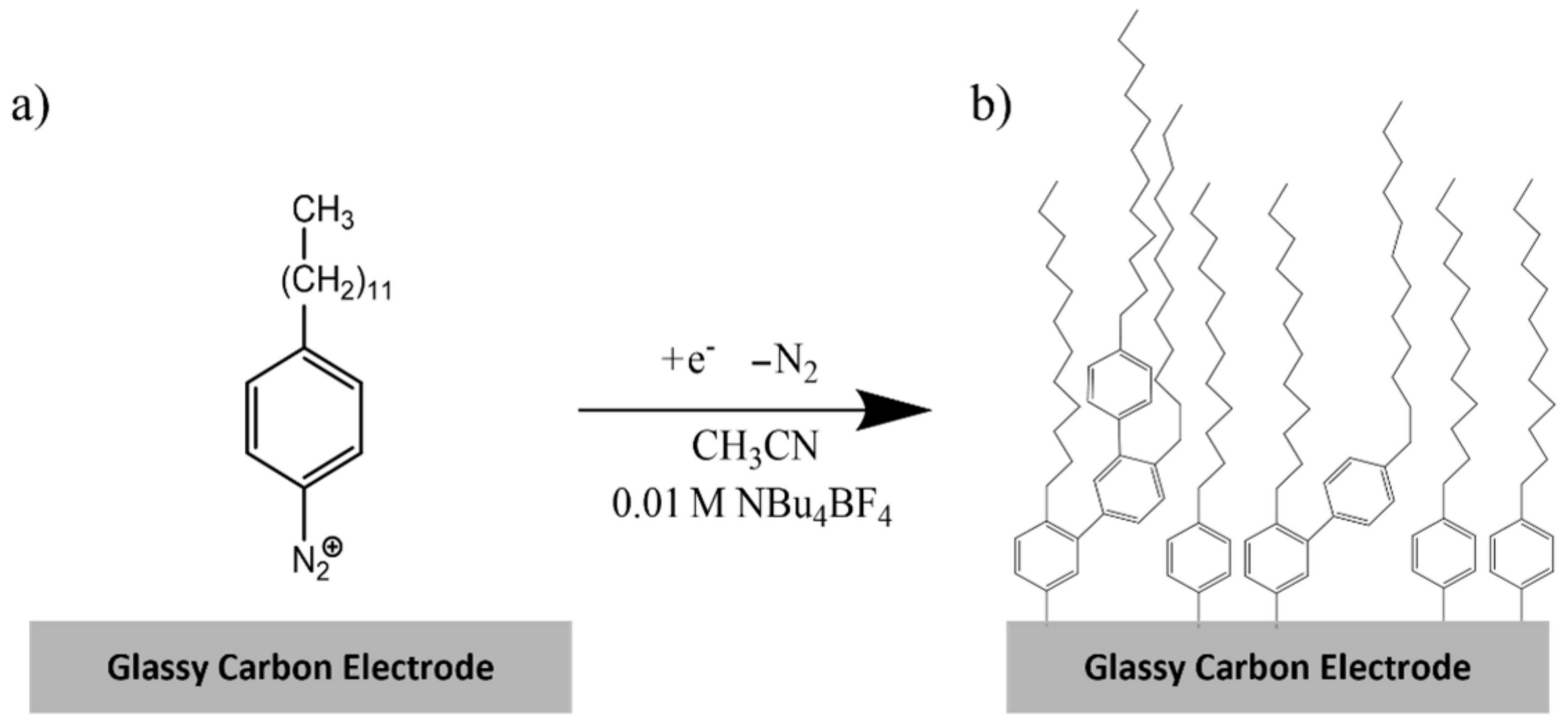
Chemical reaction for the electrodeposition of (**a**) DDAN using cyclic voltammetry (**b**) to form the first layer on the surface of a GCE.

To prepare the hybrid bilayer, DDAN and EDAN were utilized as precursors to covalently attach the alkylaryl chains onto the GCE surfaces. As described in Section 2 (also shown in Scheme S1), DDAN was synthesized, isolated, and characterized using NMR, MS, and FTIR (Figure S1–S3). EDAN was prepared in situ using 2-ethyl aniline (Scheme S2). To graft DDAN onto GCE surfaces, thirty consecutive cycles of CV were performed at a scan rate of 0.05 V s^−1^ between 0.6 V and −0.9 V vs. Ag/AgCl (Scheme 1). Every time, the HBLMs were prepared on a new, bare GCE to avoid any remnants from prior surface modifications interfering with the study. Figure 1 shows the first, second, and last two voltammograms for DDAN grafting. As shown in Figure 1, a broad irreversible peak can be seen at 0 V for the first scan, which represented the reduction and loss of N_2_ as previously reported for other diazonium modifications [50,54,58]. Our investigations showed that after thirty cycles, the current difference between the last two voltammograms displayed less than 2% difference for almost all measurements. 

### 3.2. Preparation of the Hybrid Bilayer Membrane (HBLM)

**Scheme 2:**
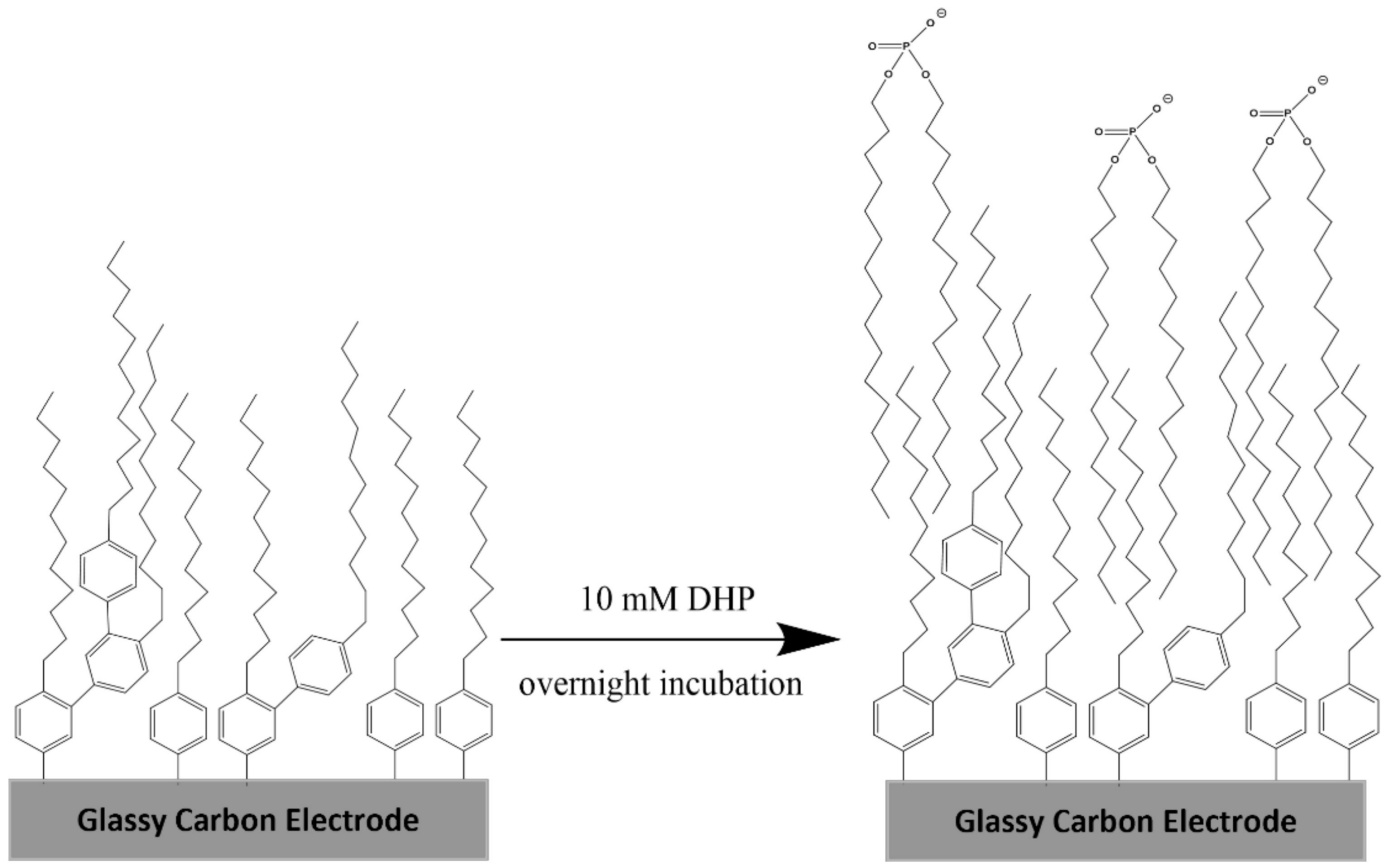
Schematic illustration of the immobilization of DHP to form the bilayer by the incubation of DHP vesicles on the GCE surface overnight.

To prepare the second layer, DHP was chosen for the electrodes with the first layer made of DDAN. For electrodes with EDAN as the first layer, BEHP was chosen as the second layer. A solution of 10 mM DHP was prepared and sonicated for 2 h to form vesicles prior to incubation. Figure S6 shows TEM images of these vesicles. Subsequently, 100 µL of the prepared solution was incubated on the DDAN-modified surfaces at room temperature overnight. The modified GCEs were then gently rinsed with deionized water followed by a 1 min sonication of the GCE-DDAN-DHP, which is coined here as the lcHBLM), to remove the non-specifically adsorbed DHP molecules/vesicles (Scheme 2).

The same procedure was performed to synthesize GCE-EDAN-BEHP using a 10 mM solution of BEHP, which is coined here as the scHBLM. Scheme 1 shows an illustration of the lcHBLM. Contact angle measurements (Table 1) performed on the bare GCE surfaces displayed approximately 73.4°. The contact angles increased after the first layer was deposited to about 85.6° due to the increased hydrophobicity resulting from the nonpolar alkyl chains populating the GCE surface for both lcHBLM and scHBLM. Following the construction of the second layer, contact angle measurements displayed a decrease to approximately 79.1° for both lcHBLM and scHBLM. This was attributed to the fact that the second layer consisted of negatively charged phosphate groups, which resulted in the increased hydrophilicity of the modified surfaces. Furthermore, ellipsometry studies were conducted to measure the length of the bilayer, which showed that the measured length was 84.8 Å for the monolayer of the lcHBLM. However, the calculated length was only 17.3 Å. We hypothesize that this is attributed to the first and second layers having different defections due to some multilayers possibly having been formed during electrografting. For future studies, the formation of multilayers can be prevented by adding a radical scavenger, such as 2,2-diphenyl-1-picrylhydrazyl, in excess or by electrodepositing diazonium moieties that possess a methyl group that is meta to the position of grafting [83,84,85]. Research in our laboratory to verify this hypothesis is in progress.

**Figure 2:**
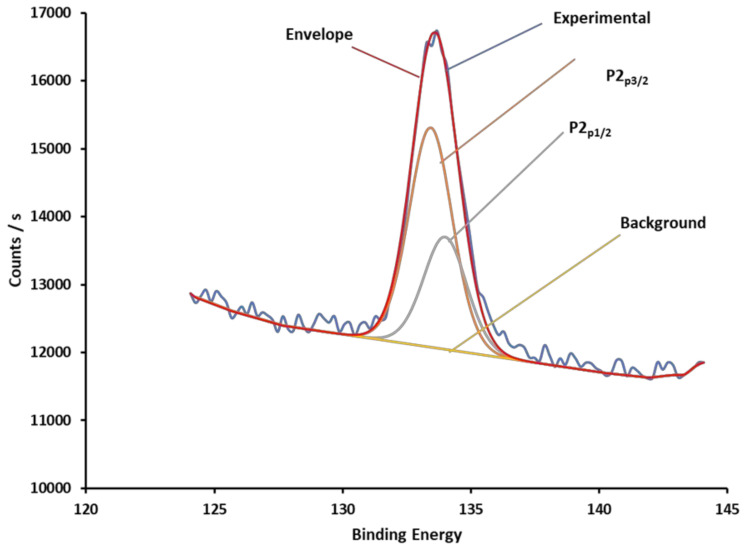
XPS spectra for the GCE-DDAN-DHP modification showing a clear distinct phosphorus peak with an asymmetric peak envelop due to the overlapping P2_p3/2_ and P2_p1/2_ spin orbit components.

**Figure 3:**
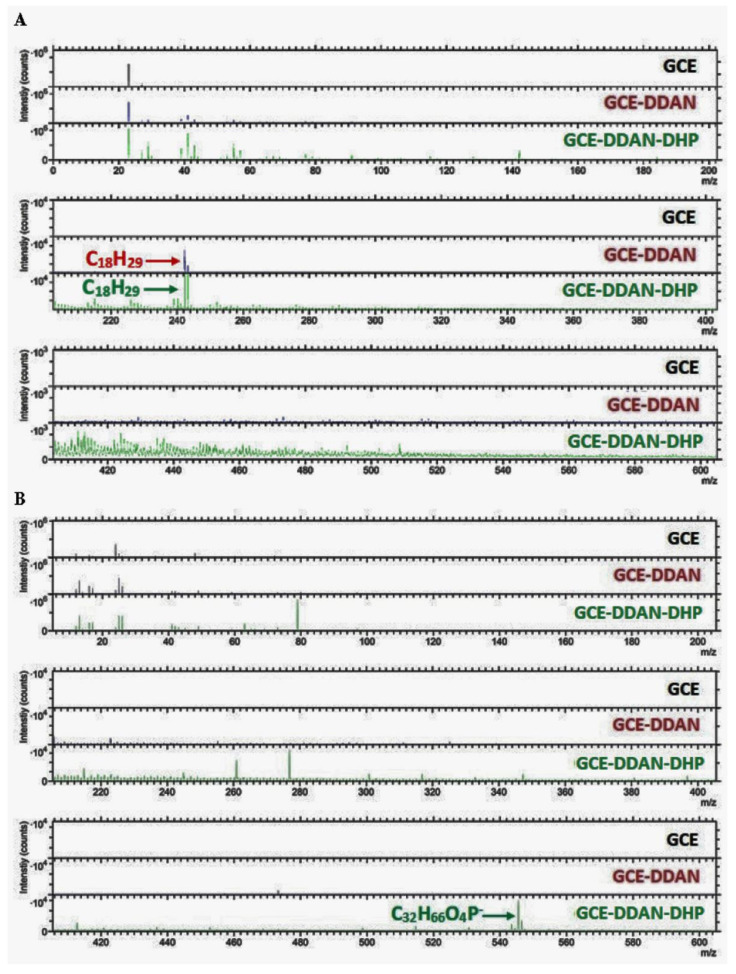
ToF-SIMS spectra for all three surfaces (500 × 500 μm^2^ area): bare glassy carbon electrode (GCE), the first layer-modified electrode (GCE-DDAN) and bilayer-modified electrode (GCE-DDAN-DHP) at negative (**A**) and positive (**B**) polarity.

Surface characterizations of the HBLMs were performed using X-ray photoelectron spectroscopy (XPS) and time-of-flight secondary ion mass spectrometry (ToF-SIMS). XPS results showed a distinctive phosphorus peak for the modification of the GCE surface with DDAN-DHP (Figure 2). ToF-SIMS data for bare GCE, GCE-DDAN, and GCE-DDAN-DHP are shown in Figure 3A,B. In Figure 3A, the spectrum with negative polarity showed the mass of 4-dodecylbenzene, which formed the first layer of lcHBLM (DDAN) for both GCE-DDAN and GCE-DDAN-DHP, and no considerable peaks for the bare GCE. The spectra with positive polarity are shown in Figure 3B with the mass of dihexadecyl phosphate (DHP; the second layer of lcHBLM) only for GCE-DDAN-DHP and no considerable peaks for bare GCE and GCE-DDAN.

### 3.3. Electrochemistry of lcHBLM- and scHBLM-Modified Surfaces

**Figure 4:**
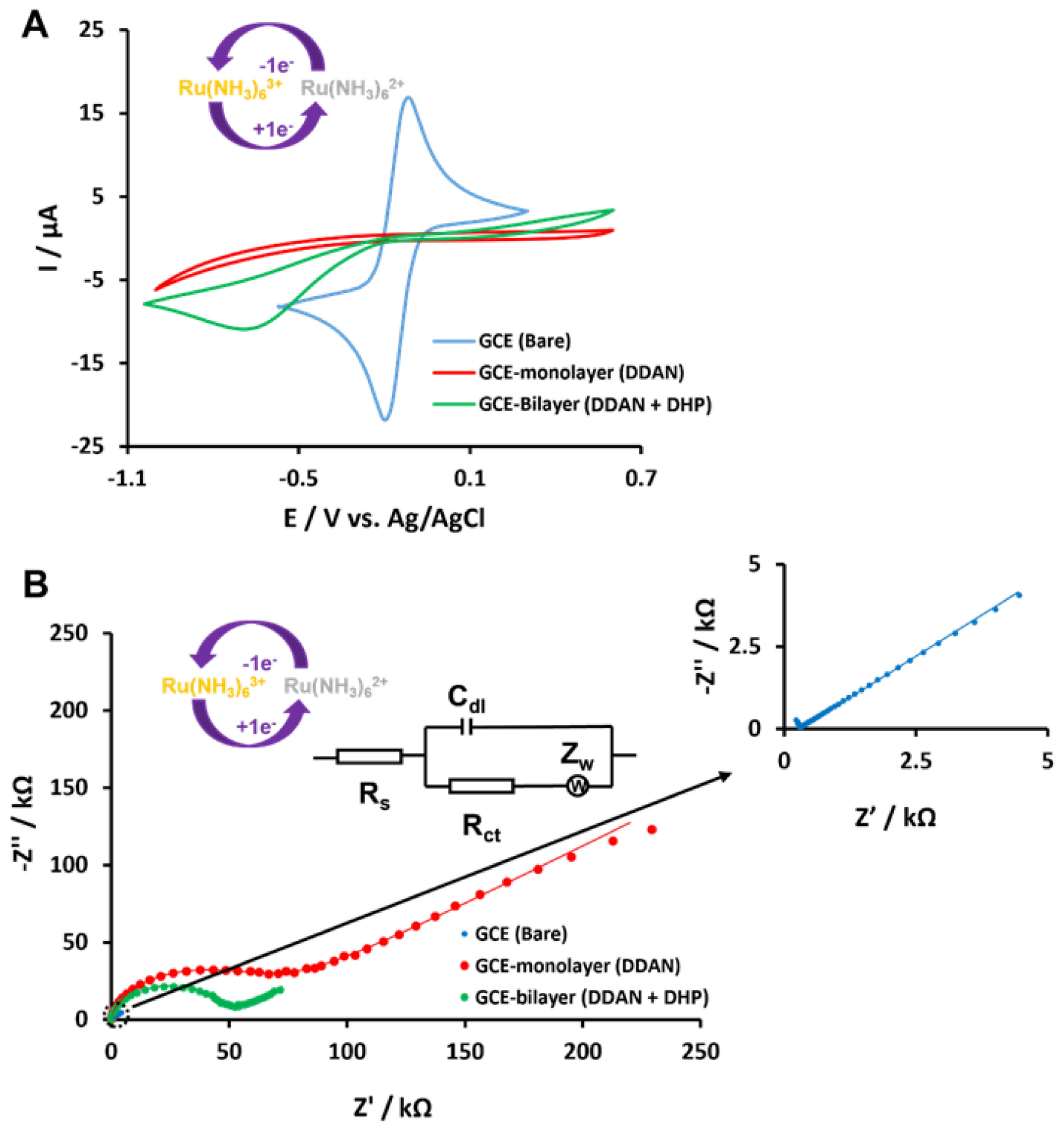

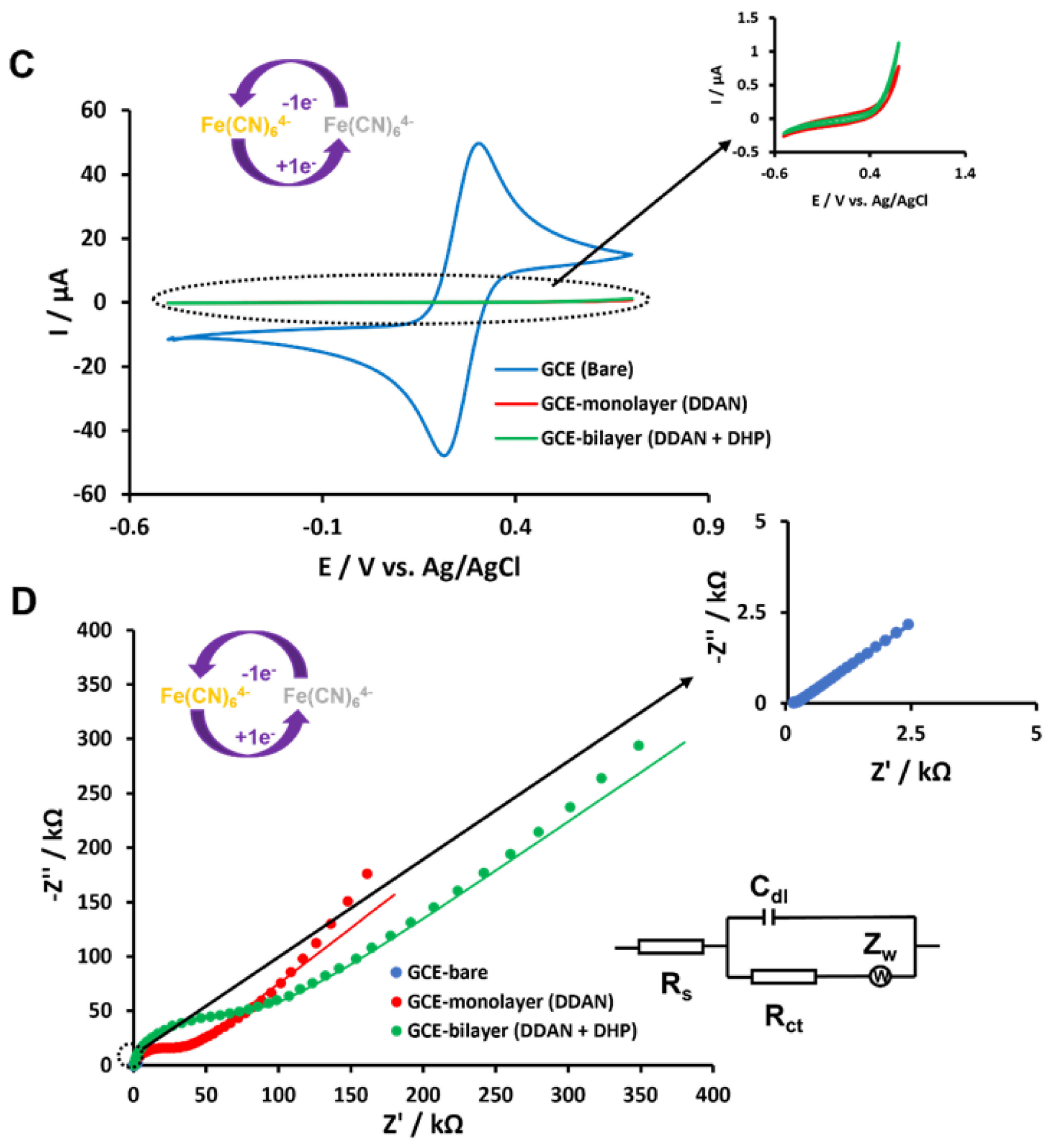
Cyclic voltammograms and Nyquist plots of the bare GCE, DDAN monolayer (the first layer), and DDAN-DHP bilayer. (**A**,**B**) show the respective CV at a scan rate of 50 mV s^−1^ and EIS using [Ru(NH_3_)_6_]^3+^ as the positively-charged redox probe. (**C**,**D**) show the respective CV and EIS spectra using [Fe(CN)_6_]^3-/4-^ as the negatively-charged redox probe. In Nyquist plots, all dots represent the experimental data and solid lines represent the simulated data with the applied frequency from 100 MHz to 100 kHz. Insets in (**B**,**D**) show the Nyquist plots obtained from bare GCEs and the modified Randle’s equivalent circuit used to fit the data. The parameters of each circuit element are included in Table S1.

Ruthenium hexamine ([Ru(NH_3_)_6_]^3+^ and ferri/ferrocyanide ([Fe(CN)_6_]^3-/4-^, having overall positive and negative charges, respectively, revealed the electrochemical characteristics of lcHBLM- and scHBLM-modified surfaces. Figure 4A shows a reversible CV (blue line) for [Ru(NH_3_)_6_]^3+^ on a bare GCE, while in the CV (red line) of the first layer (GCE-DDAN), [Ru(NH_3_)_6_]^3+^ shows no detectable peaks under the given conditions. After incubation of the second layer (DHP), a small broadened reductive peak of [Ru(NH_3_)_6_]^3+^ was observed. We attributed this phenomenon to the presence of negatively charged phosphorous groups which facilitated the diffusion of the positively charged [Ru(NH_3_)_6_]^3+^ to the surface. EIS results for the same electrode are shown in Figure 4B. The blue graph (insertion) shows the small R_ct_ of [Ru(NH_3_)_6_]^3+^ on a bare GCE. The red graph shows a relatively high R_ct_ when the electrode was modified with the first layer (GCE-DDAN). Similar to CV, EIS for [Ru(NH_3_)_6_]^3+^ in the presence of a second layer (GCE-DDAN-DHP), a decrease in R_ct_ was observed compared to the monolayer (green graph). These behaviors were reproducible for all measurements performed on different days with new solutions and different GCE surfaces. A similar behavior was observed for scHBLM (which consists of shorter molecules for both first and second layers) as shown in Figure S5.

[Fe(CN)_6_]^3-/4-^ as a negatively charged electrochemical probe showed results in a reverse trend compared to the positively charged [Ru(NH_3_)_6_]^3+^ (Figure 4C,D). CV (blue line) in Figure 4C shows the reversible voltammogram of [Fe(CN)_6_]^3-/4-^ on a bare GCE. Meanwhile, the GCE-DDAN and GCE-DDAN-DHP showed no detectable peaks under the given conditions. EIS results showed a relatively high R_ct_ of [Fe(CN)_6_]^3-/4-^ on a GCE-DDAN (red), while for [Fe(CN)_6_]^3-/4^^-^, the presence of the second layer (GCE-DDAN-DHP) added to the semicircle diameter in EIS (green), which was attributed to the repulsion of the negatively charged groups in the second layer. For scHBLM, a similar behavior was observed, which is shown in Figure S2C,D. To simulate all EIS graphs, a modified Randles equivalent circuit (Figure 4B,D) was utilized with all values of the equivalent circuit elements as summarized in Table S1.

Furthermore, when the C_dl_ of the lcHBLM in the context of [Ru(NH_3_)_6_]^3+^ (Table S1) was examined, an increase in capacitance was observed (from 48.2 to 740 nF) between the GCE-DDAN and GCE-DDAN-DHP, which was hypothesized to be due to the increase in charge presented by the negatively charged phosphate groups of the DHP and the positively charged [Ru(NH_3_)_6_]^3+^, resulting in increased charge separation. However, a slight decrease in C_dl_ was observed for [Fe(CN)_6_]^3-/4-^ (from 60.5 to 57.2 nF) indicating that the overall charge separation remained relatively unchanged. Furthermore, when the Z_w_ was examined, an increase in diffusion was observed for [Ru(NH_3_)_6_]^3+^ (from 5.81 to 38.6 μMho∙s^−1/2^), which was hypothesized to be due to the attraction of the positively charged ions to the negatively charged DHP. This was further verified by the slight decrease in Z_w_ for the [Fe(CN)_6_]^3-/4-^ (from 5.18 to 2.86 μMho∙s^−1/2^), which was thought to be due to the slightly more hindered diffusion of the probe as a result of the repulsion between the negatively charged [Fe(CN)_6_]^3-/4-^ and DHP layers.

**Figure 5:**
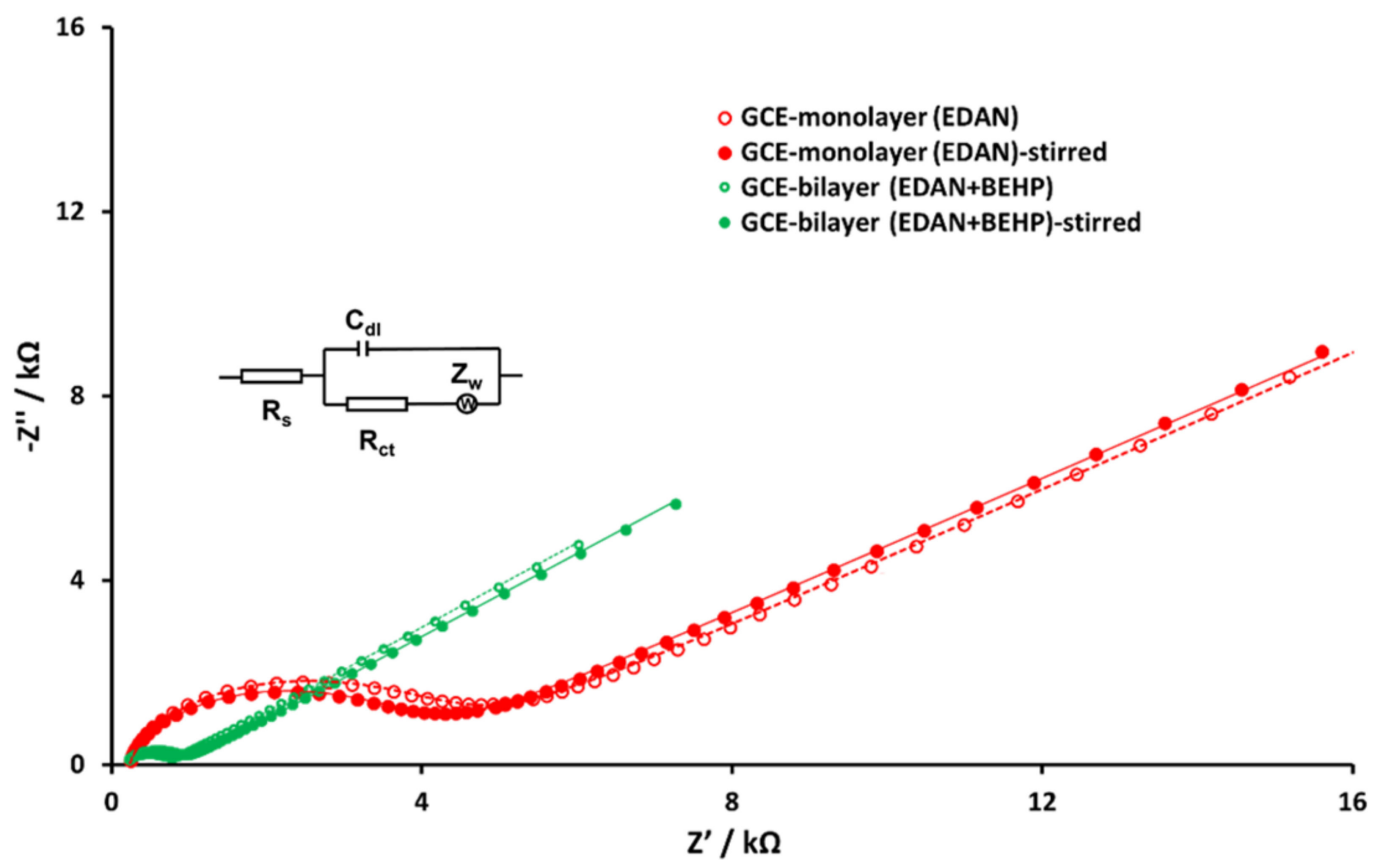
Nyquist plots of modified GCEs with monolayer (the first layer) (GCE-(EDAN); green circles), and bilayer (GCE-(EDAN+BEHP); red circles). Results are shown before (empty circles) and after (filled circles) stirring at 500 rpm for 5 min using [Ru(NH_3_)_6_]^3+^ as the positively-charged redox probe with the applied frequency ranging from 100 MHz to 100 kHz. The values of each equivalent circuit element are included in Table S2.

To investigate the mechanical stability of the bilayer, we stirred both EDAN-modified GCEs and EDAN-BEHP-bilayer-modified GCEs at 500 rpm for 5 min. EIS was performed using [Ru(NH_3_)_6_]^3+^ as the positively-charged redox probe before and after high-speed rotation (Figure 5). Values of the simulated equivalent circuit elements are shown in Table S2.

A comparison of each EIS result showed that both monolayer and bilayer were stable after a high-speed rotation. Work towards electrochemical kinetic investigations using HBLM-modified rotating disc electrodes are in progress in our laboratory.

### 3.4. Interaction with Aβ_1–42_

**Figure 6:**
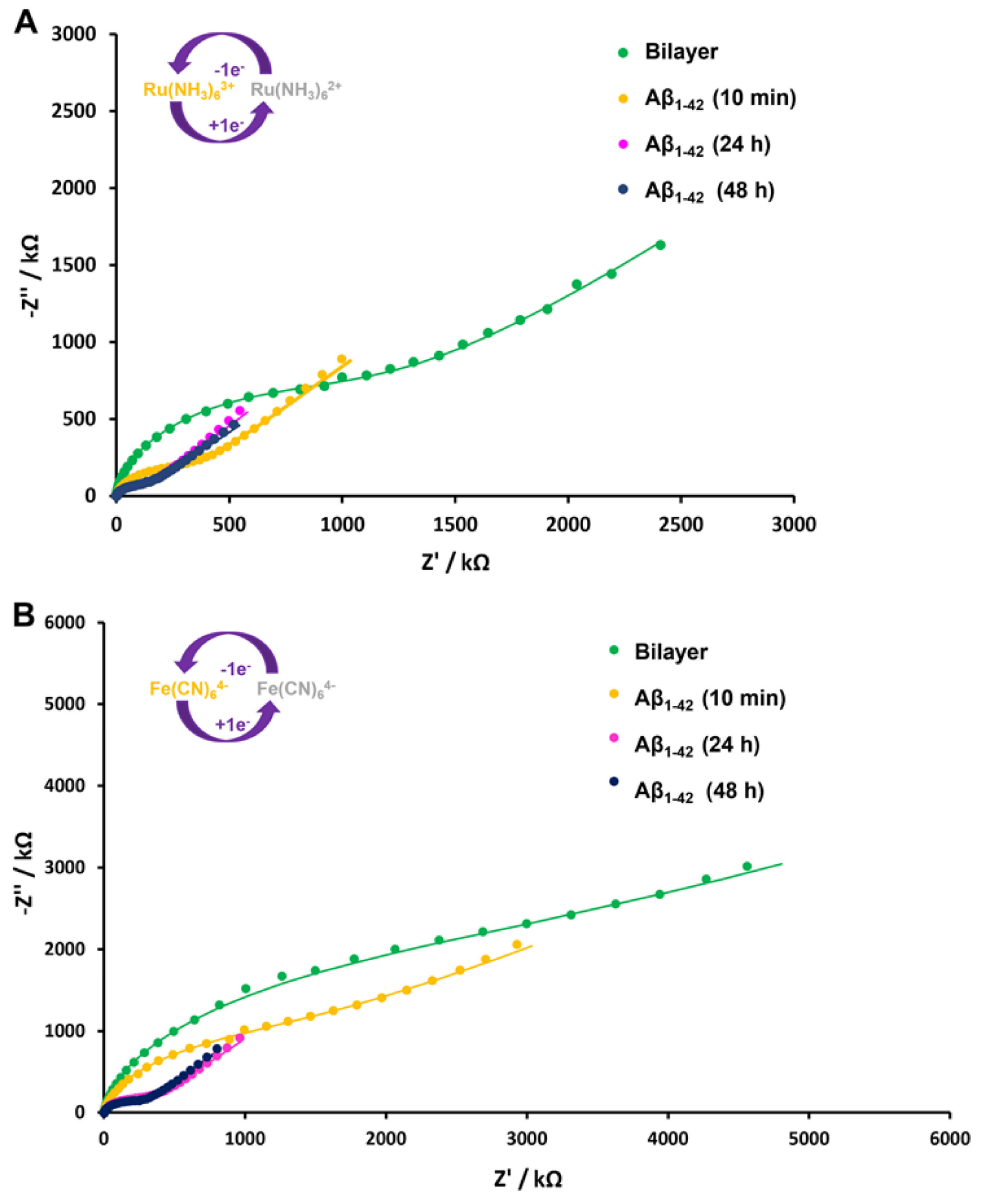
Nyquist plots of GCE-DDAN-DHP bilayer using [Ru(NH_3_)_6_]^3+^ (**A**) and [Fe(CN)_6_]^3-/4-^ (**B**) as the redox probe in the absence of Aβ_1–42_ (green), and after 10 min (yellow), 24 h (purple) and 48 h (dark blue) incubation in a solution of Aβ_1–42_. All dots represent the experimental data and solid lines represent the simulated data with the applied frequency from 100 MHz to 100 kHz. The values of each equivalent circuit element are included in Table S3.

Aβ_1–42_, which is a well-described hallmark protein of AD [71,72,73,74], was used as a model system to explore the applications of lcHBLM-modified surfaces (the lcHBLM was used as opposed to the scHBLM due to the size of the protein being too large to be embedded in the scHBLM). To study the interaction of Aβ_1–42_ with the lcHBLM, we prepared a fresh solution of 10 µM of Aβ_1–42_ in PBS (pH 7.4) and incubated an aliquot of the peptide solution (20 mL) for 10 min, 24 h, and 48 h on the lcHBLM-modified surfaces. PBS was used in this proof-of-concept study so that any electrochemical changes detected were solely attributed to the interaction of Aβ_1–42_ with the lcHBLM. In the presence of biological fluids, non-specific adsorption of interfering particles could have provided misleading results in these preliminary experiments. However, further investigations are planned to study the behaviour of Aβ_1–42_ within the lcHBLM in the presence of biological fluids such as cerebrospinal fluid. After incubation, the electrodes were washed thoroughly with PBS and then deionized water before EIS measurements (Figure 6) were taken using [Ru(NH_3_)_6_]^3+^ and [Fe(CN)_6_]^3-/4-^ as redox probes. As shown in Figure 6A,B, the R_ct_ of Aβ_1–42_-modified surfaces decreased significantly after 10 min of incubation time (purple). This decrease in R_ct_ was attributed to the aggregation of Aβ_1–42_ on the bilayer creating disruption on the ordered membrane surface that facilitated the diffusion of redox probes to the GCE surface. The disruption of neuronal cell membranes with the oligomers and fibrils of Aβ_1–42_ to form pores was previously described to cause neurotoxicity [75,76,77,78,79,80]. A significant decrease in R_ct_ was observed with a similar trend for both redox probes, despite the fact that the charges of probes were opposite. This strengthened our hypothesis that some nano/micro pores were formed in the lcHBLM layer, which might have facilitated the charge transfer between the GCE surface and redox probes, as has been reported previously [86,87]. Additionally, when other circuit elements were examined in the context of the Aβ_1–42_ experiments, both [Fe(CN)_6_]^3-/4-^ and [Ru(NH_3_)_6_]^3+^ experiments showed a consistent and gradual increase in C_dl_ with respect to incubation time ranging from 38.9 to 48.3 nF for [Fe(CN)_6_]^3-/4-^ and from 39.8 to 59.2 nF for [Ru(NH_3_)_6_]^3+^ (Table S3). This was hypothesized to be due to an increase in charge separation between the undisturbed portions of the lcHBLM and [Ru(NH_3_)_6_]^3+^ probe as Aβ_1–42_ further aggregated and settled within the biomimetic membrane. However, further experiments need to be performed to verify this hypothesis. Meanwhile, the Z_w_ for both probes showed an increase from 236 to 1330 μMho∙s^−1/2^ for [Fe(CN)_6_]^3-/4-^ and from 560 to 1930 μMho∙s^−1/2^ for [Ru(NH_3_)_6_]^3+^. Similar studies have been performed using octadecanethiol monolayers on Au electrodes as described by Valincius et al. [88], who observed an instantaneous increase in capacitance upon the injection of small Aβ_1–42_ oligomers (1–4 nm in diameter) followed by a gradual return to near original C_dl_ within an hour, which they attributed to the disruption of the octadecanethiol SAM. However, their studies were performed on a smaller time scale (1 h). Valincius et al. [89] also reported that Aβ_1–42_ oligomers caused damage to the tethered BLMs composed of varying phospholipids, which they hypothesized to be due to the Aβ_1–42_ aggregating into pore-like structures.

Further studies aiming to observe the formation of Aβ-based pores on lcHBLM-modified surfaces are in progress in our laboratory using atomic force microscopy (AFM). Another noticeable trend in EIS results was that a significant decrease in R_ct_ was observed after a short time (10 min) of incubation with Aβ_1–__42_. The difference between the R_ct_ values obtained at 24 and 48 h of incubation was not statistically significant (Table S3). In terms of differences in R_ct_ observed with [Ru(NH_3_)_6_]^3+^ as the redox probe, there was a significant decrease of 72.1% in R_ct_ between 0 and 10 min, while between 10 min and 24 h, there was a further 17.1% decrease. However, in between 24 h and 48 h incubation periods, there was a negligible decrease of 0.8% in R_ct_. In the case of the [Fe(CN)_6_]^3-/4-^ as the redox probe, there was a 26.2% decrease between 0 and 10 min. A significant decrease of 51.8% in R_ct_ was observed between 10 min and 24 h, but only a 3.8% decrease in R_ct_ was detected between 24 h and 48 h incubation periods. These observations implied that the disruption of the lcHBLM layer took place at the early stages of Aβ_1–42_ aggregation. Electrochemical investigations to discover small molecules that would affect the interaction of Aβ_1–42_ with the lcHBLM-modified surfaces is in progress in our laboratory.

## 4. Conclusions

Our preliminary results displayed the synthesis and electrografting of a novel hybrid bilayer membrane on the surface of GCEs. The lcHBLM-modified GCEs were utilized to study the Aβ_1–42_ aggregation process upon interaction with the surface-anchored membrane. This platform can be customized according to the purpose of the study by choosing appropriate molecules that are used for diazonium salts for the covalently attached first layer as well as the phospholipid (or other bipolar molecules) as the second layer. A combination of different molecules can also be used for the two layers to adjust the affinity, thickness, and other physical properties of the HBLMs. We envisage that similar EIS studies can be performed using lcHBLM-modified GCES in connection with membrane-bound biomolecules to understand their interactions with small molecules in drug screening assays. Furthermore, future studies aim to use these novel HBLMs to quantify biomolecules in the context of biological fluids by embedding antibodies, aptamers, as well as other biorecognition elements within the HBLM.

## Figures and Tables

**Table 1:** Contact angle measurements of the bare GCEs, GCEs modified with the DDAN layer, and the GCEs with the HBLM (DDAN + DHP) with the average contact angle values obtained from six independent GCE surfaces (*n* = 6).

Electrode	Contact Angle Measured (°)	Electrode	Contact Angle Measured (°)	Electrode	Contact Angle Measured (°)
Bare GCE 1	75.864	GCE-DDAN 1	89.482	GCE-DDAN-DHP 1	80.702
Bare GCE 2	66.880	GCE-DDAN 2	85.151	GCE-DDAN-DHP 2	80.441
Bare GCE 3	71.702	GCE-DDAN 3	83.490	GCE-DDAN-DHP 3	81.125
Bare GCE 4	75.156	GCE-DDAN 4	84.299	GCE-DDAN-DHP 4	75.250
Bare GCE 5	73.963	GCE-DDAN 5	86.724	GCE-DDAN-DHP 5	78.196
Bare GCE 6	76.869	GCE-DDAN 6	86.220	GCE-DDAN-DHP 6	79.021
**Average Contact Angle (°), *n* = 6**	**73.405**		**85.894**		**79.122**

## Supplementary Materials

The following supporting information can be downloaded at: https://www.mdpi.com/article/10.3390/mi13040574/s1, Scheme S1: Schematic diagram for synthesis of DDAN; Scheme S2: Schematic diagram for in situ synthesis of EDAN; Figure S1: Mass spectrometric characterization of DDAN; Figure S2: FTIR spectra for (A) DDAN and (B) the precursor amine (4-dodecylaniline); Figure S3: NMR spectrum of DDAN; Figure S4: Cyclic voltammograms for the electrodeposition of EDAN; Figure S5: Cyclic voltammograms and Nyquist plots of bare GCE, GCE-EDAN layer, and GCE-EDAN-BEHP bilayer; Figure S6 TEM images of a solution of 10 mM of DHP at (A) 0.5 μm magnification and (B) 1 μm magnification. Table S1: The values of simulated equivalent circuit elements of Nyquist plots shown in Figure 4B,D; Table S2: The values of simulated equivalent circuit elements of Nyquist plots shown in Figure 5 with the redox probe [Ru(NH_3_)_6_]^3+^; Table S3: The values of simulated equivalent circuit elements of Nyquist plots shown in Figure 6.
