# Supplementary material for: Electrografting a Hybrid Bilayer Membrane via Diazonium Chemistry for Electrochemical Impedance Spectroscopy of Amyloid-β Aggregation
Source: Micromachines (Basel). 2022 Apr 5;13(4):574. doi: 10.3390/mi13040574 (PMC9029378; doi:10.3390/mi13040574)
Supplement: Supplementary file 1 [file micromachines-13-00574-s001.zip › micromachines-1623482-supplementary.pdf]

Supplementary Information

for

Electrografting a Hybrid Bilayer Membrane via Diazonium Chemistry for Electrochemical Impedance Spectroscopy of Amyloid-β Aggregation

Hamid Fini[†], Qusai Hassan[†], Meissam Noroozifar and Kagan Kerman[*,††]

Department of Physical and Environmental Sciences, University of Toronto Scarborough, 1265 Military Trail, Toronto, M1C 1A4, ON, Canada; h.fini@utoronto.ca (H.F.); qusai.hassan@mail.utoronto.ca (Q.H.); m.noroozifar@utoronto.ca (M.N.)
* Correspondence: kagan.kerman@utoronto.ca (K.K.)
† These authors contributed equally.
†† This paper is dedicated to the memory of Dr. Ari Chow and Professor Ian R. Brown.

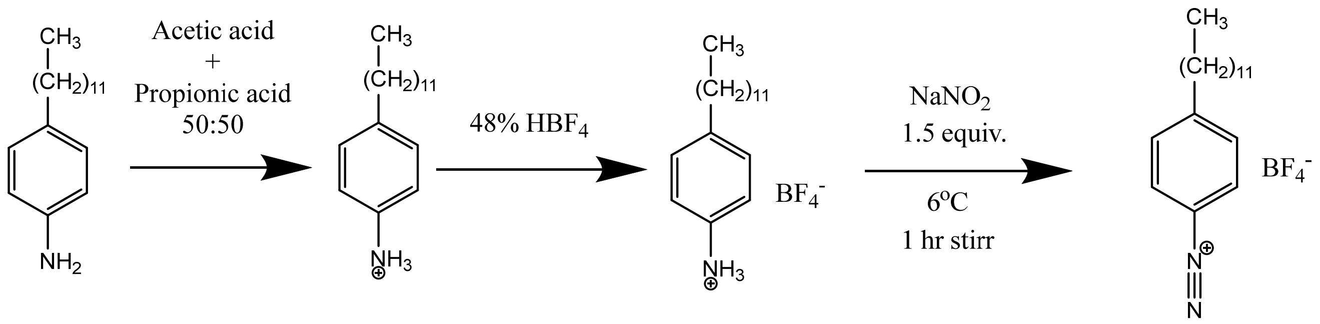

Scheme S1. Schematic diagram for synthesis of 4-dodecylbenzenediazonium tetrafluoroborate (DDAN).

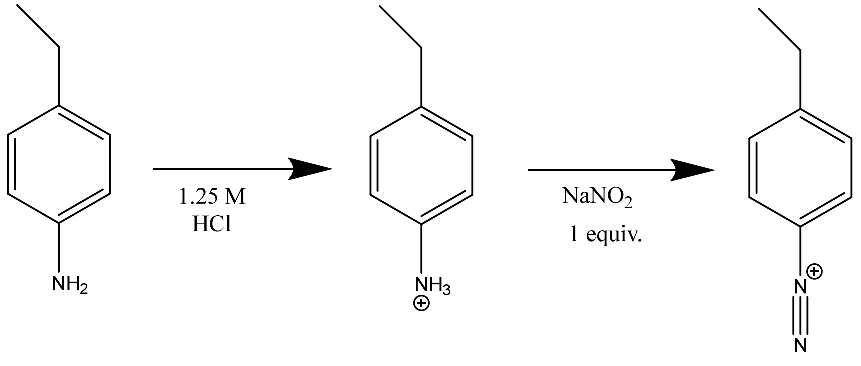

Scheme S2. Schematic diagram for *in situ* synthesis of 4-ethylbenzene diazonium (EDAN). As described in the Experimental section, 2 mM ethyl aniline was dissolved in 1.25 M HCl followed by the addition of 1 equivalent of $NaNO_2$.

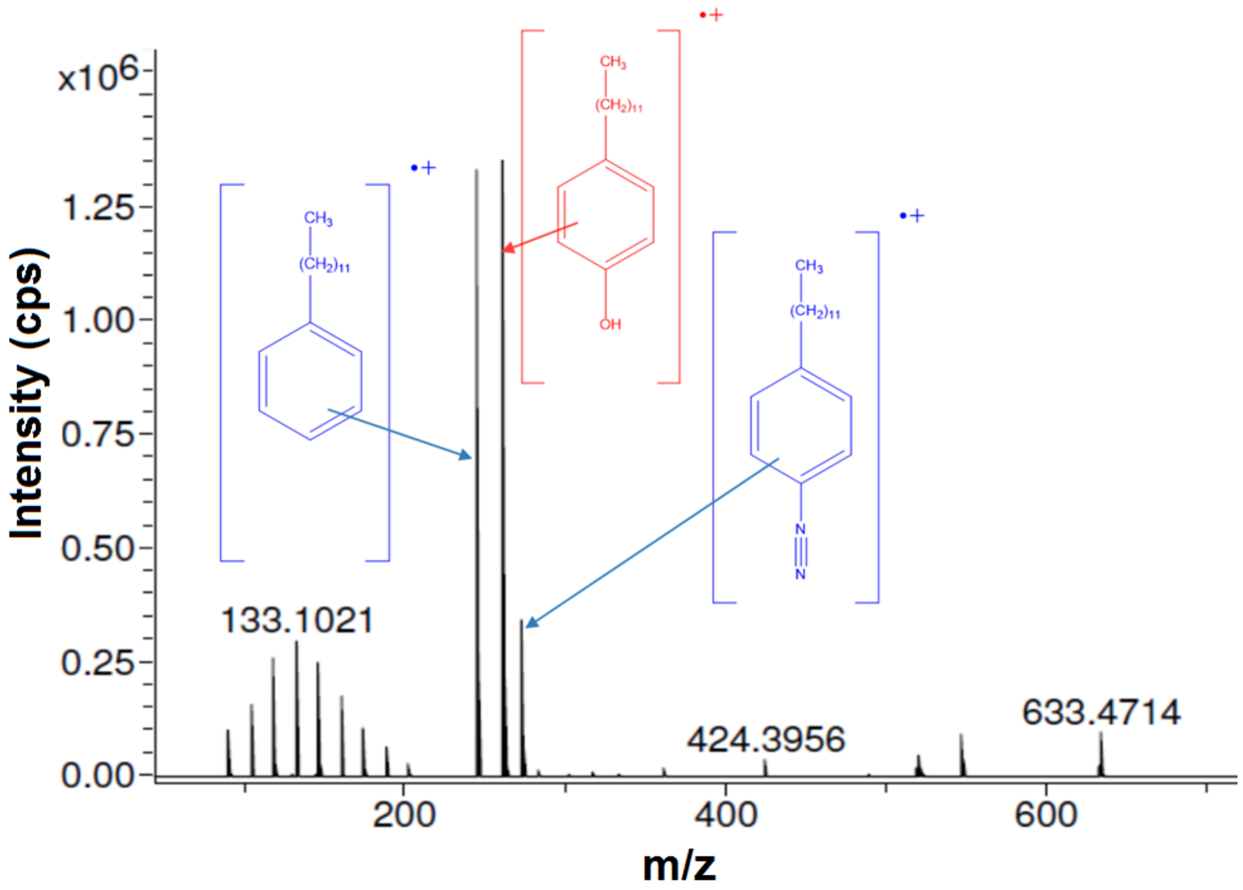

Figure S1. Mass spectrometric characterization of the synthesized diazonium salt (DDAN, 4-dodecylbenzene-diazonium tetrafluoroborate).

A

B

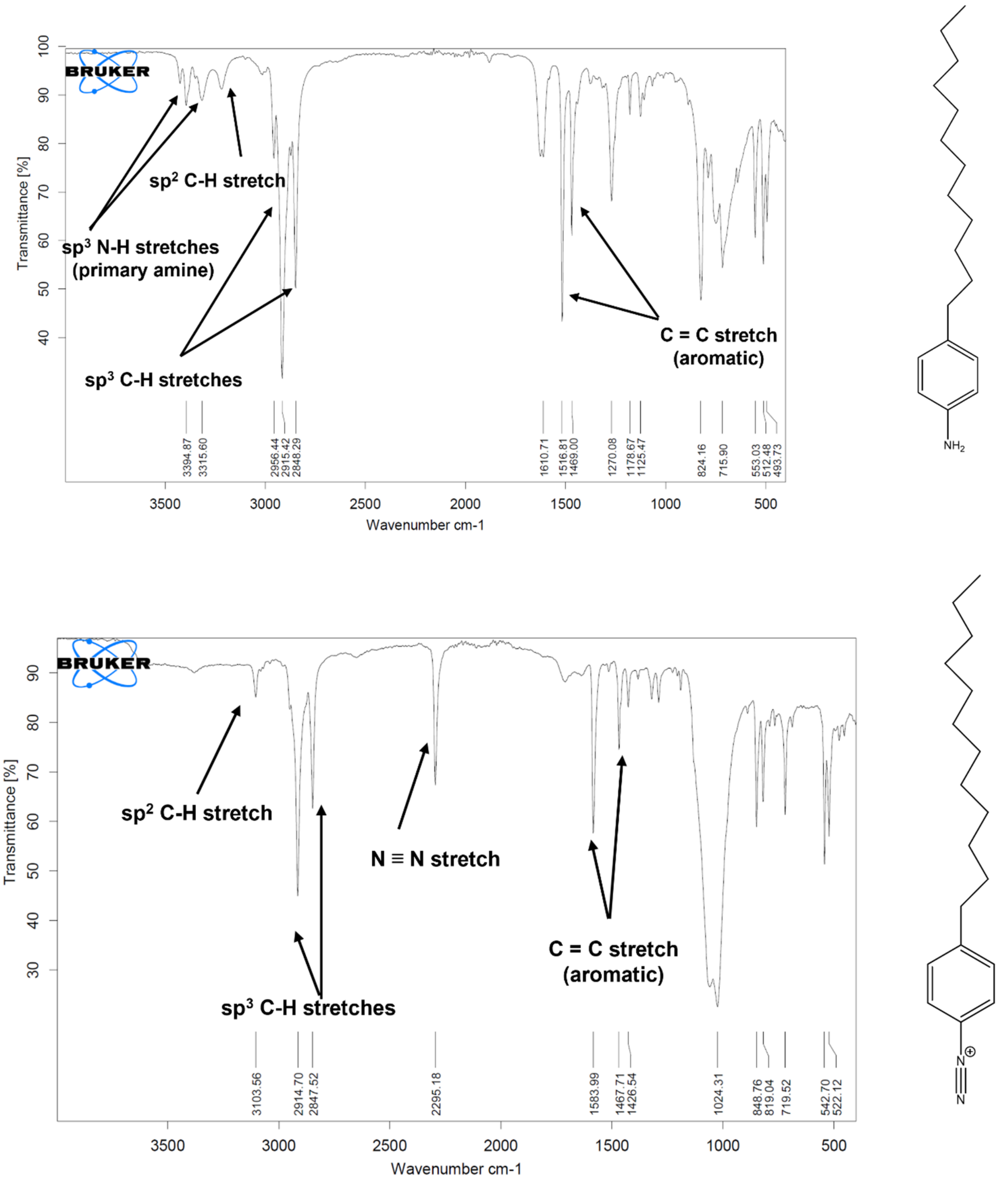

Figure S2. FT-IR spectra for the (A) synthesized diazonium salt (DDAN, 4-dodecylbenzene diazonium tetrafluoroborate) and (B) the precursor amine (4-dodecylaniline).

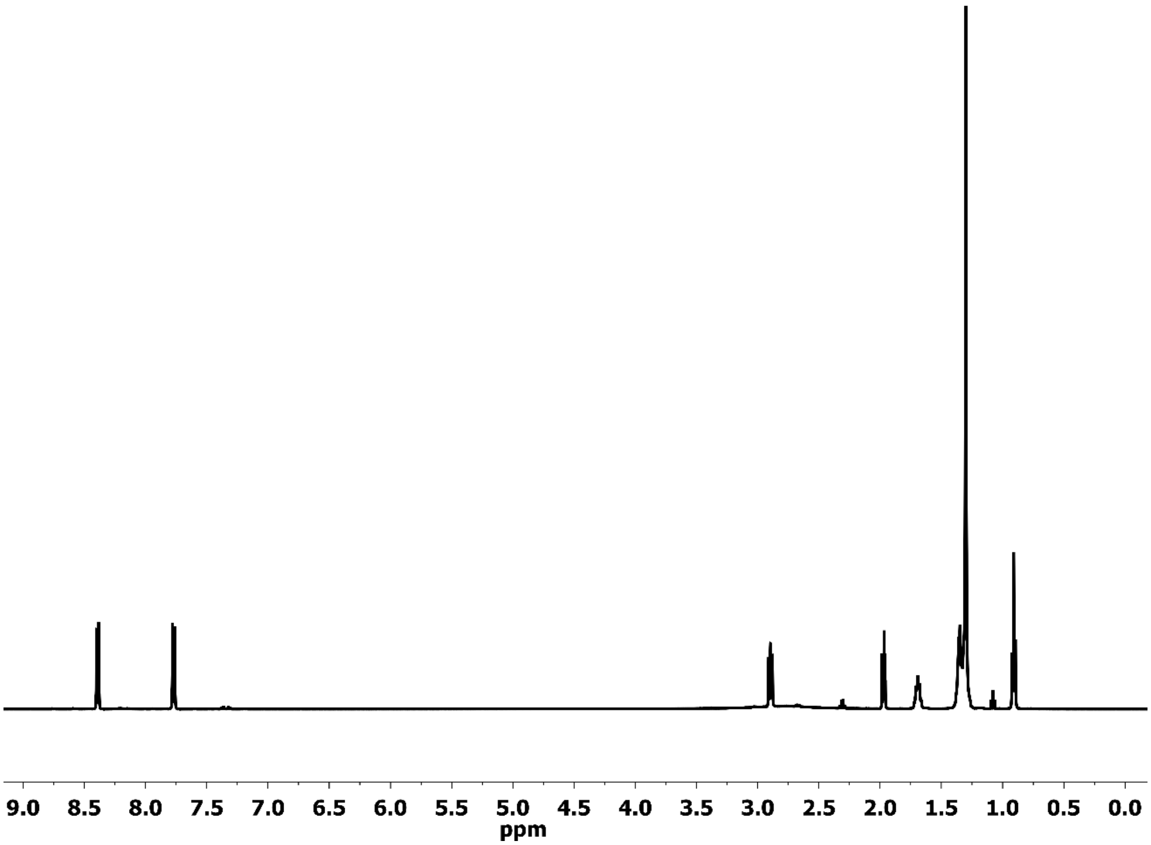

Figure S3. NMR spectrum of the synthesized diazonium salt (DDAN, 4-dodecylbenzene diazonium tetrafluoroborate)

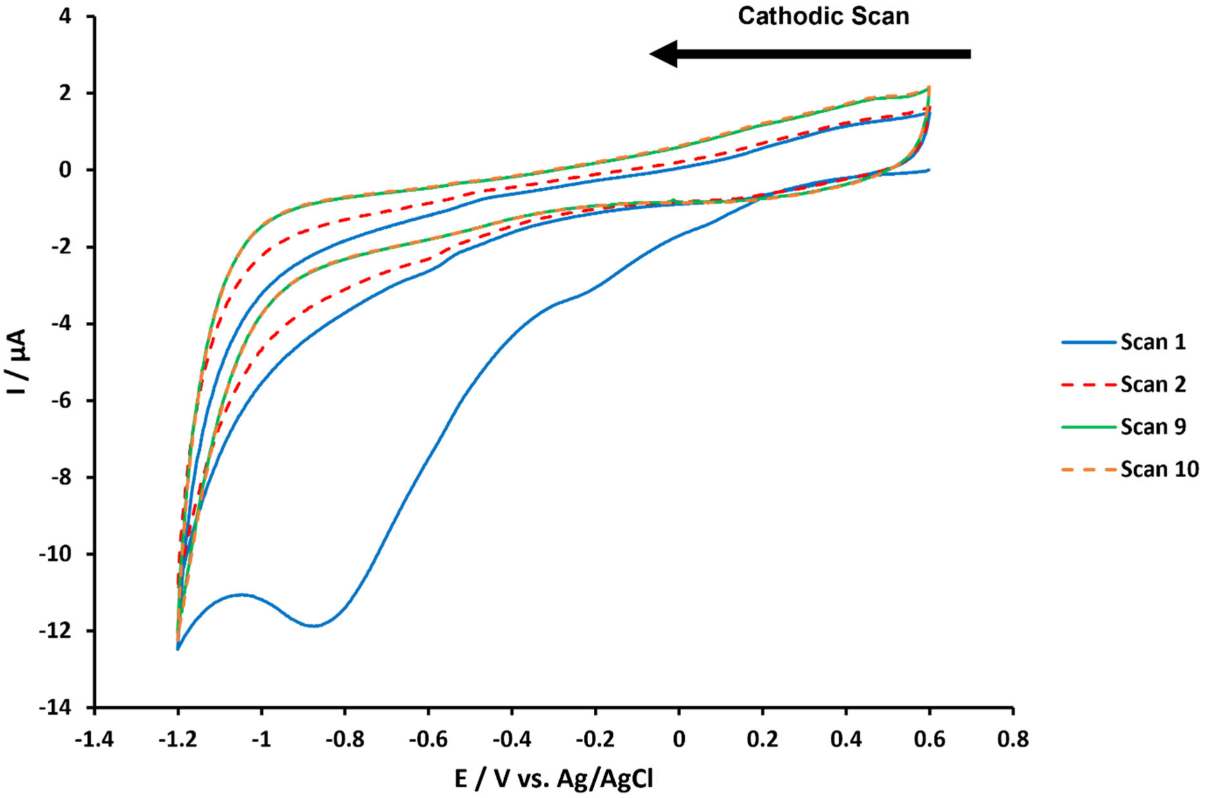

Figure S4. Cyclic voltammograms for the electrodeposition of 4-ethylbenzenediazonium (EDAN) on GCE surface at a scan rate of 50 mV s^{-1}.

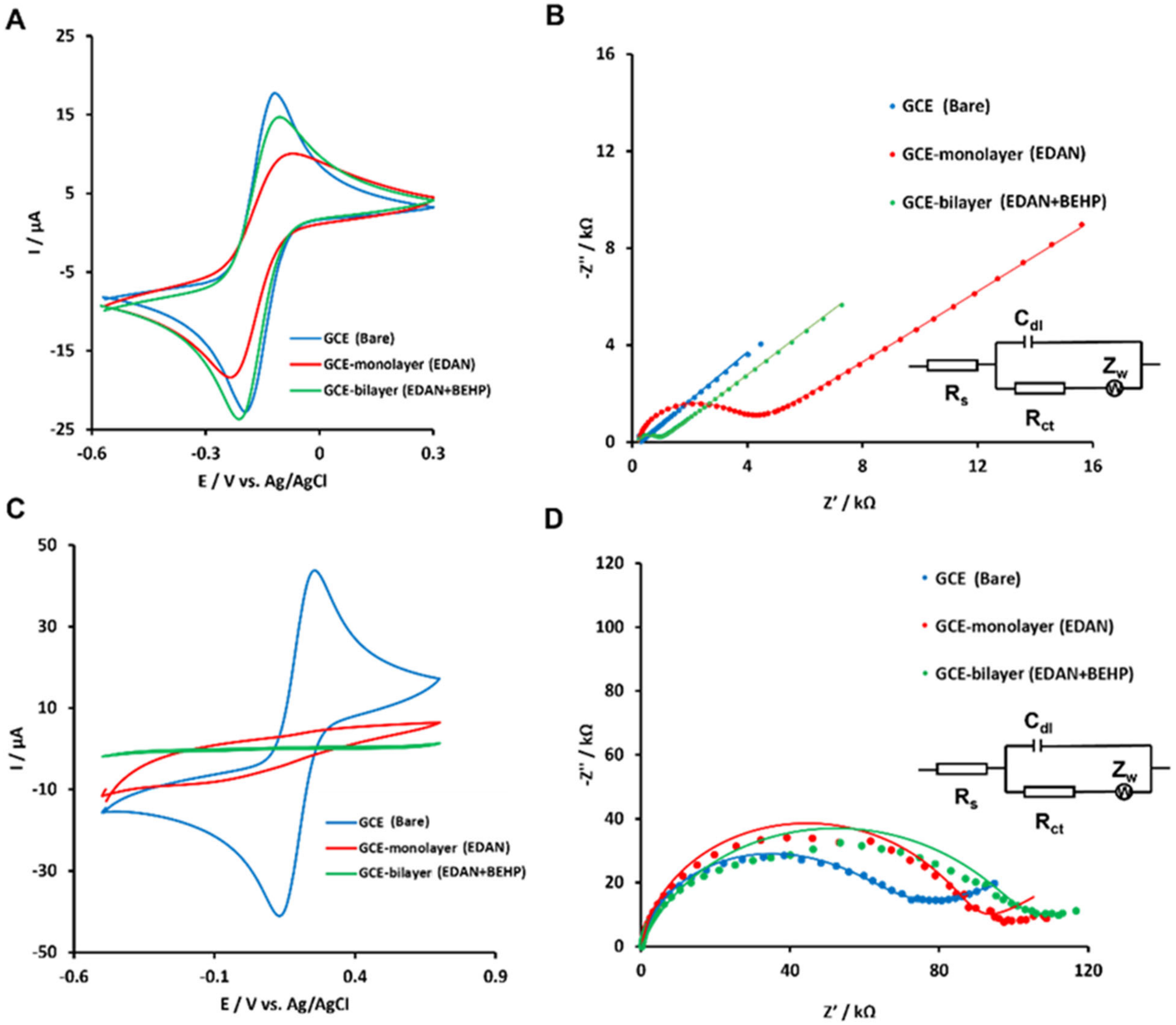

Figure S5. Cyclic voltammograms and Nyquist plots of bare GCE, GCE-EDAN layer, and GCE-EDAN-BEHP bilayer (scHBLM)-modified GCEs. (A) and (B) show the respective CV and EIS spectra using $[Ru(NH_3)_6]^{3+}$ as the redox probe. (C) and (D) show the respective CV and EIS spectra using $[Fe(CN)_6]^{3-/4-}$ as the redox probe. In Nyquist plots, all dots represent the experimental data and solid lines represent the simulated data at an applied bias of -0.20 V and +0.25 V vs Ag/AgCl at frequencies between 100 MHz and 100 kHz.

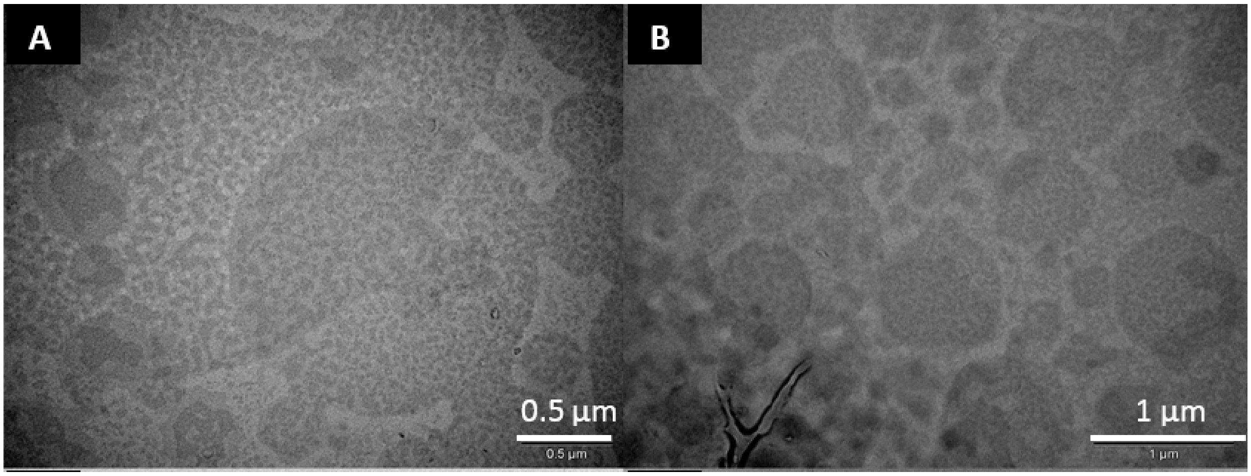

Figure S6. TEM images of 10 mM DHP vesicles at (A) 0.5 µm magnification and (B) 1 µm magnification.

Table S1. The values of simulated equivalent circuit elements of Nyquist plots as shown in Figures 4B and 4D.

Electrochemical probe	Surface modification	R_s	R_{ct}	C_{dl}	N	Z_w	M
$[Ru(NH_3)_6]^{3+}$	Bare GCE	40	0.262	4.45	1	215	0.5
	DDAN	232	55.6	48.2	0.946	5.81	0.415
	DDAN-DHP	210	48.4	740	0.919	38.6	0.46
$[Fe(CN)_6]^{3-/4-}$	Bare GCE	123	0.148	4.2	0.413	260	0.525
	DDAN	189	30	60.5	0.935	5.18	0.51
	DDAN-DHP	188	67.3	57.2	0.943	2.86	0.473

Table S2. The values of simulated equivalent circuit elements of Nyquist plots as shown in Figure 5 with the redox probe $[Ru(NH_3)_6]^{3+}$.

Surface modification	Stirring time	R_s	R_{ct}	C_{dl}	N	Z_w	M
**GCE-EDAN**	No stirring	235	3.38	156	0.892	80.8	0.404
**(Monolayer)**	1 min	232	3.82	155	0.892	71.9	0.404
**GCE-EDAN-BEHP**	No stirring	176	754	498	0.766	145	0.466
**(Bilayer)**	1 min	171	588	638	0.744	174	0.472

Table S3. The values of simulated equivalent circuit elements of Nyquist plots as shown in Figure 6.

Electrochemical probe	Incubation time	R_s	R_{ct}	C_{dl}	N	Z_w	M
$[Ru(NH_3)_6]^{3+}$	**0**	193	1020	39.8	0.952	560	0.493
	**10 min**	181	285	46.9	0.935	1070	0.526
	**24 h**	173	110	58.8	0.915	1740	0.531
	**48 h**	204	102	59.2	0.915	1930	0.495
$[Fe(CN)_6]^{3-/4-}$	**0**	196	1320	38.9	0.960	236	0.352
	**10 min**	183	974	46.7	0.945	392	0.415
	**24 h**	197	291	49.2	0.937	1120	0.552
	**48 h**	204	240	48.3	0.941	1330	0.565
